# Incubation phase transmission of foot-and-mouth disease virus in cattle: experimental evidence and simulated impacts

**DOI:** 10.1038/s41598-025-34132-x

**Published:** 2026-01-07

**Authors:** Carolina Stenfeldt, John M. Humphreys, Jonathan Arzt

**Affiliations:** 1 Foreign Animal Disease Research Unit, U.S. Department of Agriculture-Agricultural Research Service, Plum Island Animal Disease Center, Greenport, NY USA; 2 College of Veterinary Medicine, Department of Diagnostic Medicine/Pathobiology, Kansas State University, Manhattan, KS USA; 3 Foreign Animal Disease Research Unit, U.S. Department of Agriculture-Agricultural Research Service, National Bio and Agro-Defense Facility, Manhattan, KS USA

**Keywords:** Foot-and-mouth disease virus, FMDV, FMD, Modeling, Transmission, Cattle, Computational biology and bioinformatics, Diseases, Microbiology

## Abstract

The capacity of any pathogen to transmit from infected hosts prior to the development of clinical disease substantially impacts the ability to effectively control an outbreak. Foot-and-mouth disease virus (FMDV) is known for its rapid spread and ability to cause severe disease outbreaks amongst susceptible livestock species. In this current investigation, it was demonstrated that cattle infected with FMDV were capable of transmitting infection at least 24 h prior to the development of clinical signs. Additionally, the progression of infection in cattle exposed to infected donors during the early infectious phase was slower than in cattle exposed at later time points, suggesting a dose-dependent effect on infection dynamics in contact-exposed cattle. To quantify the impact, outcomes from the transmission experiment were used to parameterize agent-based simulations at three biological levels, within-host, within-herd, and between-farm. Simulations revealed that outbreaks spread more rapidly and infect more cattle and farms when models account for preclinical transmission. Specifically, including pre-clinical transmission in a between-farm simulation resulted in a 33.7% increase in the number of affected farms, demonstrating that incubation phase infectiousness has important implications for outbreak preparedness and response.

## Introduction

Foot-and-mouth disease (FMD) is a viral disease of cloven-hoofed livestock renowned for its ability to spread rapidly amongst susceptible animals. The causative agent, foot-and-mouth disease virus (FMDV), an *Aphthovirus* within the *Picornavirus* family, exists as seven distinct serotypes with multiple strains and subtypes within each serotype^1,2^. The clinical characteristics of FMD in naïve cattle involve varying degrees of lameness and hypersalivation associated with development of vesicular lesions in areas of non-haired skin, including the oral cavity and feet as well as udders. This may be accompanied by transient fever, reduced feed intake, and reduced milk production in lactating animals^3,4^. Animals vaccinated against FMD may become subclinically infected following virus exposure (neoteric infection). Although such individuals typically shed substantially less virus than clinically affected animals, they may still function as a source of contagion and disease propagation. The potential impact of subclinical infections for FMD control and transmission biology underscores the importance of investigating incubation phase contagion.

Understanding the timing of infectiousness relative to the onset of clinical signs is crucial for controlling FMD outbreaks. Like other infectious diseases, the clinical phase of FMD is preceded by an incubation phase^5,6^. The duration of the incubation phase varies depending on multiple factors, such as the route, frequency, and duration of virus exposure, intrinsic characteristics of the virus strain, the quantity or dose of virus transmitted, and the susceptibility of the exposed host in terms of natural or acquired immunity. For FMDV infection in cattle, the duration of the incubation phase has been reported to be 2–7 days for controlled experimental studies^7–9^. However, this range is likely wider under field conditions due to the broader variability of exposure conditions. The virus quantities shed in oronasal secretions gradually increase through the incubation phase to subsequently peak during the clinical phase of the disease^10–12^. The duration of the latent phase, the period from the time of infection to the onset of infectiousness, is rarely directly observed or quantified in experimental or model-based simulation studies. However, the onset of infectiousness is believed to correlate strongly with increasing virus shedding^7,13^.

An FMDV transmission study done in pigs demonstrated the occurrence of a preclinical infectious phase lasting at least 24 h; thus, the duration of the latent phase was at minimum 24 h shorter than the incubation phase^14^. It was further shown that the existence of this preclinical infectious phase had a significant impact on models of FMD outbreaks in the U.S. pig production system^5^. Similarly, it is believed that FMDV-infected sheep with either mild or no clinical signs, contributed to the massive dissemination of infection during the early stages of the 2001 FMD epidemic in the U.K.^15,16^.

Reports of experiments investigating FMDV transmission in cattle are less consistent. Orsel et al*.* concluded that preclinical transmission was substantial^17^. An earlier investigation by Graves et al*.,* using a 1:1 donor: recipient ratio with a contact duration of 24 h, reported that infected cattle could transmit FMDV up to 48 h before the appearance of lesions^18^. In contrast, Charleston et al*.,* using a closely related virus strain, concluded that the onset of infectiousness occurred, on average, 0.5 days after the first appearance of vesicular lesions^13^. These differences were likely caused by differences in study design: in the experiment reported by Orsel et al*.,* animals were continuously housed together in groups, whereas the experiment conducted by Charleston et al*.* was based on time-limited (8 h) exposure of one infected animal to one naïve recipient. The different outcomes of these studies emphasize the critical importance of study design in interpreting experimental outcomes. The limited number of FMDV incubation phase transmission studies in cattle, varied study designs, and inconsistent findings regarding infectiousness onset highlight the need for further research using methodologies that capture natural behaviors and interactions among cattle.

To address this need, the current investigation combined an empirical animal experiment with an agent-based modeling (ABM) approach to expand the knowledge of preclinical FMDV transmission in cattle. The study’s objective was to determine the onset of infectiousness while maintaining the behavioral patterns of cattle housed in groups, reflecting common livestock management practices. The empirical study involved 18 Holstein heifers of which two were experimentally infected with FMDV and used to sequentially expose four groups of four naïve animals through consecutive 24 h time slots. The outcome of this empirical experiment demonstrated that infected cattle could transmit FMDV at least 24 h before the appearance of vesicular lesions, confirming the occurrence of preclinical transmission. Moreover, evidence suggested that the quantity of virus shed by the infecting donors influenced the response and disease progression in the recipient animals. ABM simulations reinforced the empirical observations of preclinical transmission, provided quantitative insights into the factors influencing the onset and duration of infectiousness, and enabled precise comparison between outbreaks with and without preclinical transmission. The combined empirical and modeling approach enhances available characterization of FMDV transmission dynamics in cattle, particularly the role of preclinical infectiousness. It offers valuable information for epidemiological modeling and developing effective control strategies.

## Results

### Experimental FMDV-transmission study

#### Donor cattle

**Fig. 1:**
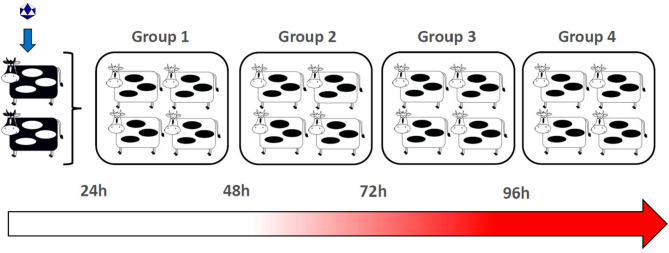
Study design. Schematic of animal study design. Two “donor animals” (dark colored animals) were infected with FMDV on time point 0. The infected donor cattle were sequentially moved through separate isolation rooms, comingling with four groups of four contact cattle each, through 24 h exposure periods. Animals subject to contact exposure were held as isolated groups and monitored for signs of foot-and-mouth disease through 7 to 14 days after completed exposure.

**Fig. 2:**
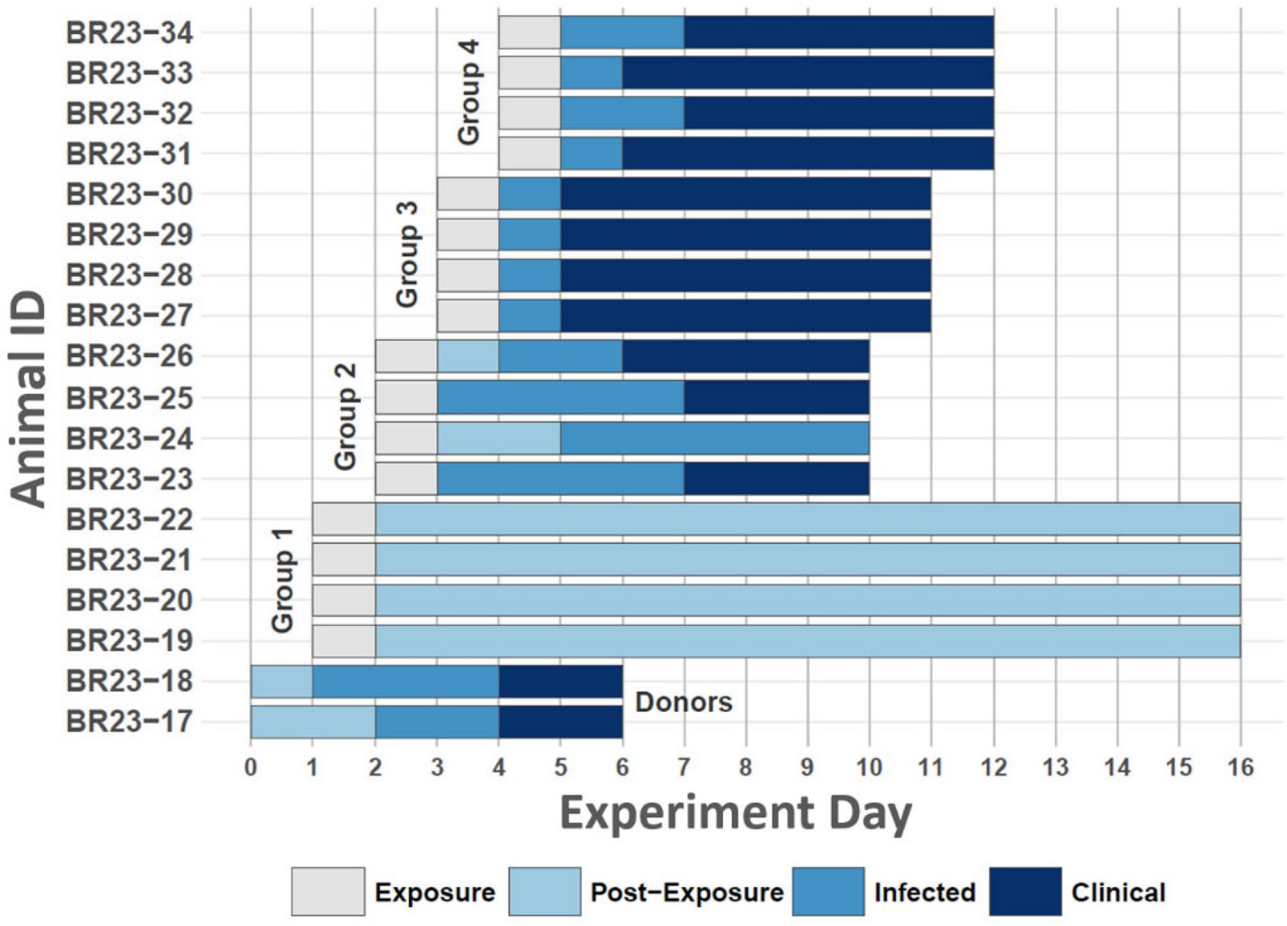
Summary of animal-level infection dynamics. Figure summarizes individual animal progression of infection from inoculation or contact exposure to experiment termination. Vertical axis lists animal identification number, and horizontal axis details timeline from study start (day 0). Animal donor or contact group designation is provided in annotated text in main plot area. Bars corresponding to individual animals are color-coded according to legend at bottom center to indicate period of contact exposure (gray), post-exposure (light tone blue), infection as evidenced by FMDV RNA-positive nasal swab or serum sample (medium tone blue), or FMD lesion detection (dark tone blue). Note that infection was not detected in Group1, animal BR23-24 did not progress to clinical disease, and period durations varied by animal.

**Fig. 3:**
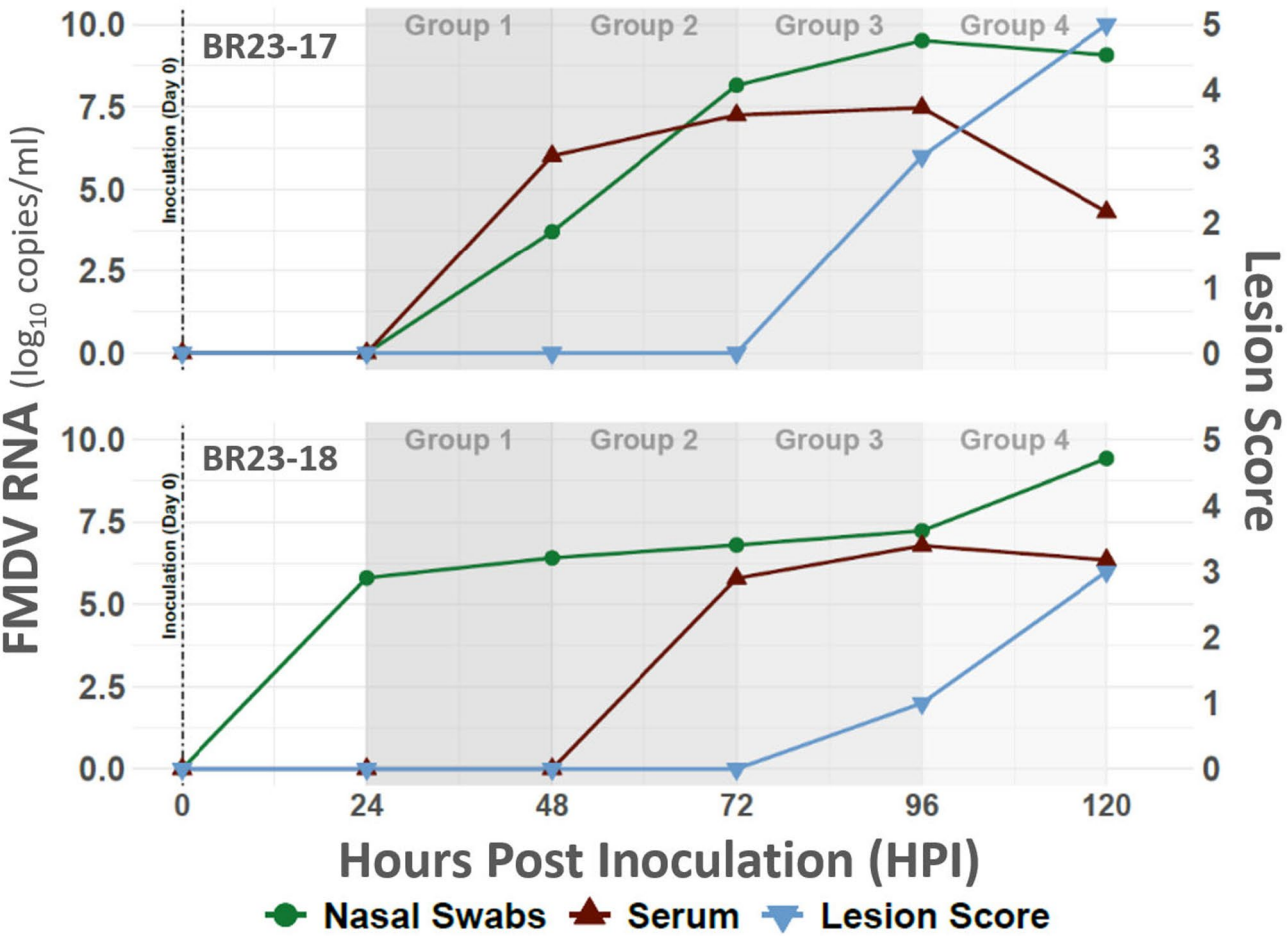
Within-host infection dynamics and lesion scores in donor animals. Figure depicts FMDV RNA quantities detected in samples from study donor animals and lesion scores documented during clinical examinations. Vertical axes at left indicate FMDV RNA quantity, and horizontal axis lists hours post donor inoculation (time 0). Secondary vertical axis at right reflects lesion score (range 0 -5). Donor animal BR23-17 is shown in top panel, and donor BR23-18 shown in the bottom panel. Trajectories for nasal swab (green circles), blood serum (red, triangles), and lesions score (blue inverted triangles) are color-coded according to legend at bottom center. Portions of background are shaded in gray color to highlight periods of animal group contact exposure by donors.

Two of a total of 18 experimental cattle were randomly selected as ‘donors’ and were infected with FMVD A24 Cruzeiro through intra-nasopharyngeal (INP) deposition of inoculum at time point 0 of the experiment. The two donor cattle were repeatedly moved into a new isolation room housing four naïve cattle, at 24 h intervals, until a total of four groups of contact cattle had been exposed for 24 h each (Fig. 1). The first clinical signs of FMD in the INP -inoculated donor cattle were observed at 96 hpi, corresponding to the transition between exposure of contact groups 3 and 4 (Figs. 1, 2 and 3). These signs consisted of fever (> 40 °C) and small vesicular lesions in the oral cavities of both animals, with additional vesicles found in the interdigital clefts of two feet of one individual (ID BR23-17, Figs. 2 and 3). Neither of the donors had any signs of lameness or hypersalivation at the first detection of vesicles. The clinical signs in the two donor animals had progressed by the termination of the final contact exposure (120 hpi), to include mild lameness and visible salivation. At that time, one individual (BR23-17) had vesicular lesions on all four feet, whereas the other donor (BR23-18) had lesions on two feet (Fig. 3). FMDV RNA was detected in nasal swabs starting at 24 h post infection (hpi) in one of the donors (BR23-18), and from 48 hpi in the other animal (Fig. 2). Viremia, defined as detection of FMDV RNA in serum, was detected at 48 hpi in BR23-17, and at 72 hpi in animal BR23-18 (Fig. 3).

#### Contact group 1

**Fig. 4:**
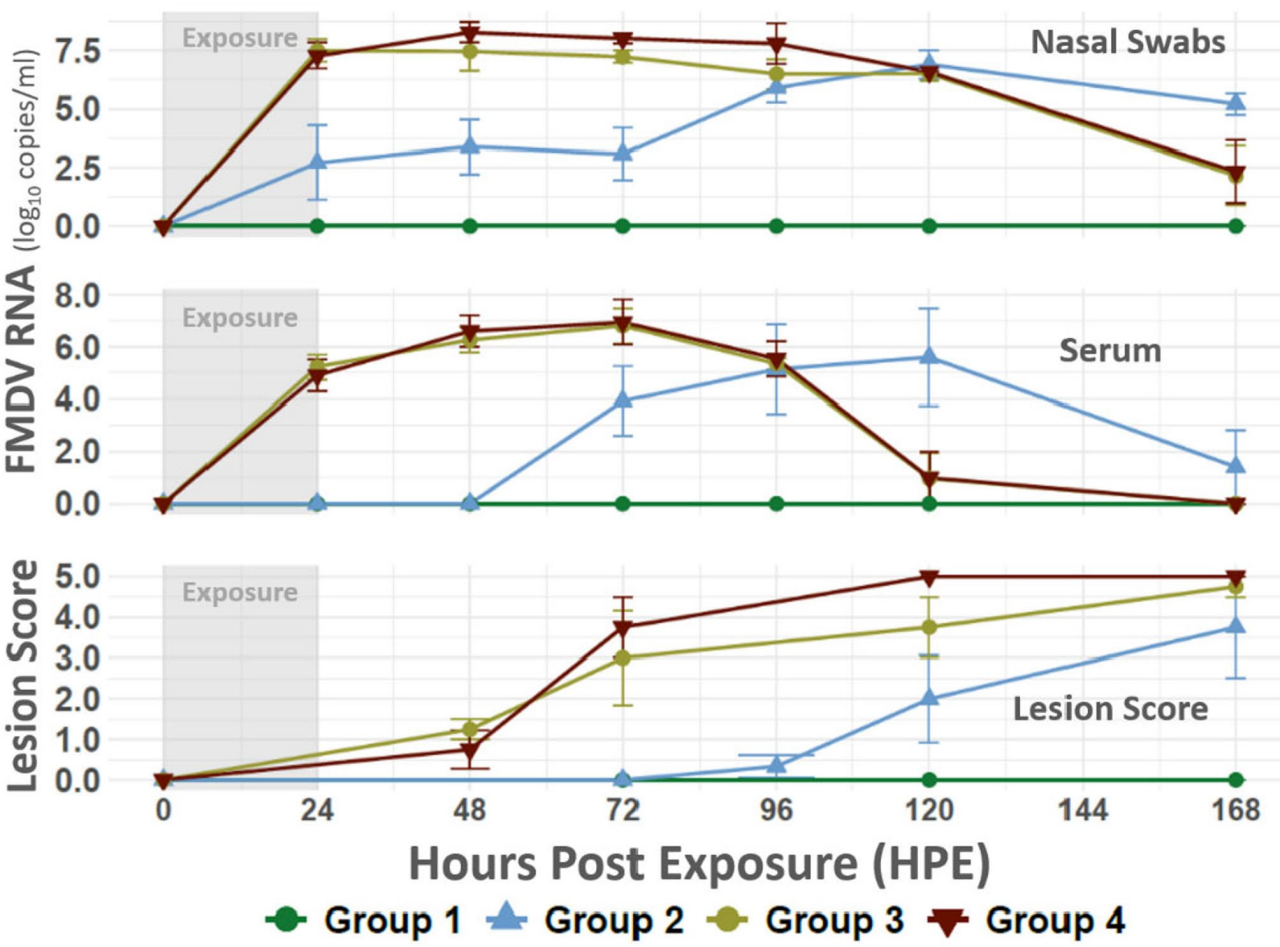
Contact animal within- host virus dynamics and lesion scores. Figure depicts FMDV RNA quantities sampled from study contact animals and lesion scores documented during clinical examination. Vertical axes at left indicate FMDV RNA quantity (top and center panels) and lesion score (bottom panel). Horizontal axis lists hours post donor exposure. Smooth lines symbolize group means with error bars providing standard deviations.

The four heifers in contact group 1 were co-housed with the two donor cattle from 24–48 hpi relative to the donors. None of the animals in contact group 1 developed any signs of clinical FMD, and all samples collected, including oropharyngeal fluid (OPF) samples obtained at 10 and 14 dpe, were negative for FMDV RNA and infectious virus (Fig. 4). Additionally, none of the animals had seroconverted by day 14.

#### Contact group 2

Contact group 2 was exposed to the donors from 48–72 hpi, during which time the donors had not yet developed clinical signs of FMD. All four individuals in contact group 2 were confirmed to be infected, but with variability in the onset of clinical disease. Nasal shedding of FMDV RNA was detected from 24 h post exposure (hpe) in two individuals, from 48 hpe in one individual, and from 72 hpe in the fourth animal (Fig. 4). FMDV RNA was detected in serum from 72 hpe in all three animals that developed clinical FMD, and at 7 dpe in the one animal that remained subclinical (Fig. 2). The earliest observed signs of clinical FMD were an oral vesicle in one animal at 96 hpe and fever (> 40 °C) in two different animals. At 5 dpe, the two animals with fever on the day prior had also developed vesicles in the mouth and/or feet (Fig. 4). The fourth animal had not developed lesions by 7 dpe when the whole group was euthanized based on animal welfare concerns.

#### Contact group 3

Contact group 3 was exposed to the donors from 72–96 hpe. The two donor cattle were subclinical at the start of the contact exposure but had developed mild FMD lesions by the end of the exposure period. High levels of FMDV RNA were detected in both serum and nasal secretions from all four contact-exposed animals by the end of the exposure period at 24 hpe (Fig. 4). All four animals in contact group 3 had oral FMD lesions at 48 hpe, corresponding to 24 h after the end of the contact exposure period (Fig. 4). Two of the animals had reached full lesion score by 72 hpe, and three of the four animals had reached full lesion score by 7 dpe (Fig. 4). The animals were euthanized at 7 dpe.

#### Contact group 4

The final contact group was exposed to the donors from 96 to 120 hpi, at which time the donors had developed clinical FMD. Similar to contact group 3, high quantities of FMDV RNA were detected in both nasal swabs and serum from all four animals by the end of the contact exposure (24 hpe). FMD lesions were observed at 48 hpe in two animals and at 72 hpe in the remaining two individuals, and all four animals had reached full lesion score by 7 dpe, at which time the animals were euthanized.

### Statistical analysis and mathematical modeling

#### Clinical parameters: virus shedding, viremia, and lesion scores

The repeated measures ANOVA revealed significant effects for nasal virus (RNA) quantity, serum virus (RNA) quantity, and lesion scores among the different contact groups over time.

Nasal Virus Quantity: There were significant main effects of group (F(2, 9) = 9.22, *p* = 0.0066) and days post-exposure (dpe) (F(6, 54) = 33.99, *p* < 0.0001), as well as a significant interaction between group and days post-exposure (F(12, 54) = 5.53, *p* < 0.0001). Specifically, animals in groups exposed to infected donors later in the donor infectious period had higher FMDV RNA quantities at earlier dpe (Figs. 3 and 4), consistent with a dose-dependent response to increased donor shedding. This interaction supports the hypothesis that later exposure coincided with higher donor virus output, leading to more rapid or intense infection dynamics in contact animals.

Serum Virus Quantity (viremia): There was no significant main effect of group on serum FMDV RNA quantity (F(2, 9) = 2.79, *p* = 0.114), indicating that average serum viral loads did not differ across groups. However, there was a significant main effect of dpe (F(6, 54) = 25.18, *p* < 0.0001) and a significant group by dpe interaction (F(12, 54) = 7.95, *p* < 0.0001). This indicates that serum FMDV quantities changed significantly over time and that the pattern of these changes differed among the groups (Figs. 3 and 4).

Lesion Scores: Significant main effects were observed for group (F(2, 9) = 5.86, *p* = 0.023) and dpe (F(6, 54) = 45.87, *p* < 0.0001), along with a significant group by dpe interaction (F(12, 54) = 3.04, *p* = 0.0025). These findings demonstrate that lesion severity differed among groups and progressed significantly over time, with varying progression patterns by group (Figs. 3 and 4). The significant interaction indicates that some groups developed lesions more rapidly or severely than others at specific time points.

#### Duration of infection phases

**Fig. 5:**
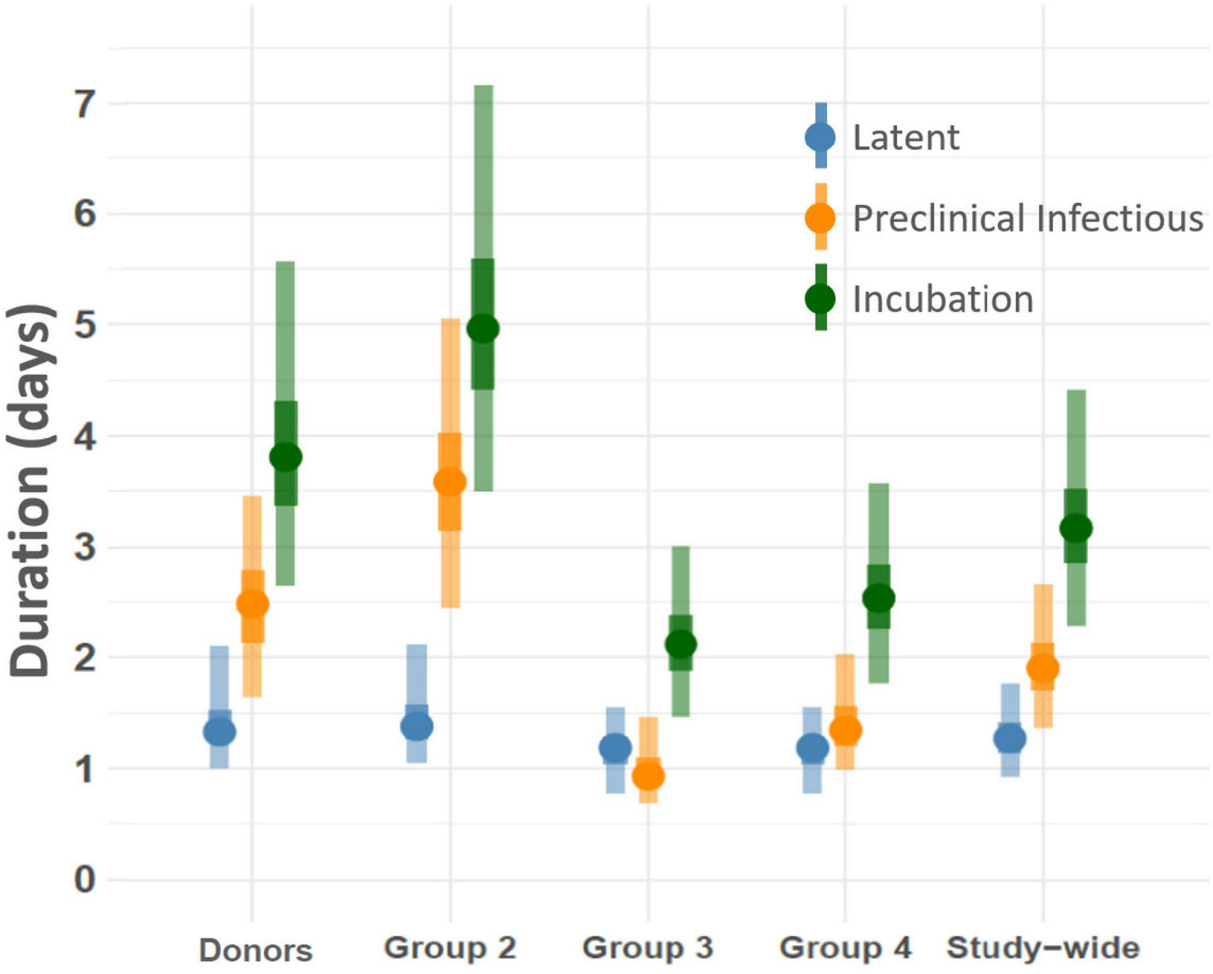
Infection phase duration estimates. Figure displays median and 95th percentile for AFT model estimated phase durations. Vertical axis at left lists time duration as measured in days, horizontal axis partitions estimates by experiment group and provides an average across all groups (Study-wide). Points symbolize median values with intersecting bars indicating the 2.5th and 97.5 percentiles at the lowest and highest extents, respectively. Darker-toned bars demarcate the 25th and 75th percentiles at the lowest and highest extent, respectively. Shapes are color-coded according to inset legend to distinguish latent, preclinical infectious, and incubation phases.

Survival models estimated the durations of the latent, subclinical, and incubation phases across groups and study-wide averages (Fig. 5). The median latent phase duration differed among groups, reflecting variability in time elapsed from infection to infectiousness. Study-wide estimates provide aggregate trends, while the 95th percentile ranges indicate within-group variability, possibly influenced by viral dose differences as a consequence of the different exposure to the donors.

**Fig. 6:**
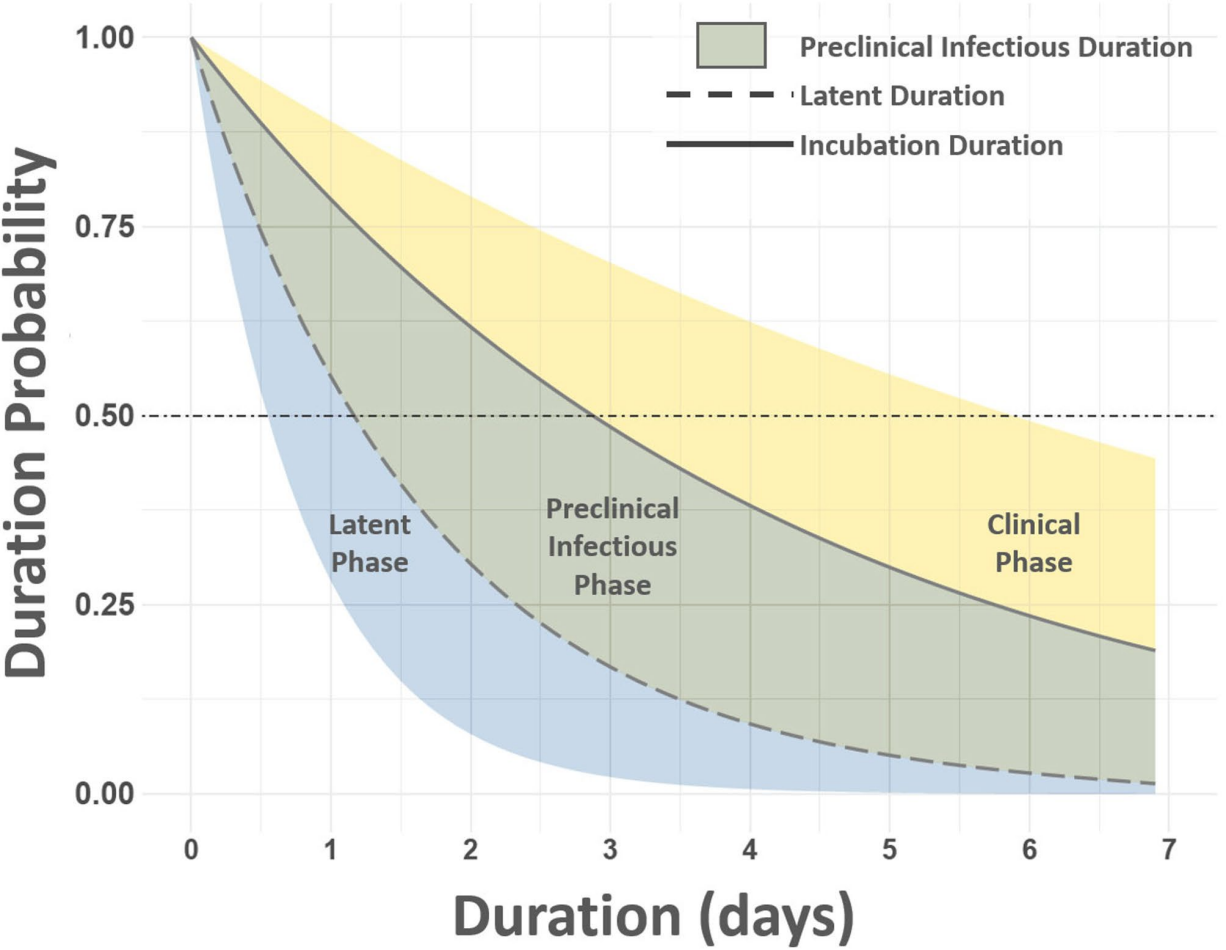
Preclinical infectious phase duration probability curve. Figure displays the probability of phase durations between 0 and 7 days. Vertical axis at left lists probability (range 0.00–1.00), horizontal axis gives durations measured in days. Horizontal dot-dash line extending from 0.50 indicates the median duration probability. Curved dashed line represents median latent phase duration and solid line depicts median total incubation period. Note that the preclinical infectious phase duration is between 1.20 and 2.90 days.

The preclinical infectious phase, when animals are infectious but before development of clinical signs, also varied among groups. Shorter durations in groups exposed later in the study may suggest that exposure to effectively higher viral doses accelerates the clinical progression, while longer durations of the subclinical infectious period indicate prolonged undetected infectiousness, which is critical for transmission dynamics. The probability of preclinical infectious phase durations from 0 to 7 days is shown in Fig. 6. The median durations of this period fall between 1.2 and 2.9 days. Probability curves for latent and incubation periods illustrate phase duration distributions within the population, offering insights into variability and transmission potential through time (Fig. 6).

The incubation phase, encompassing both latent and subclinical infectious phases, differed in duration between groups. Shorter incubation periods in groups exposed relatively later in the study may reflect higher initial viral exposure, leading to faster disease progression.

#### Relationship between exposure dose and progression of infection

**Fig. 7:**
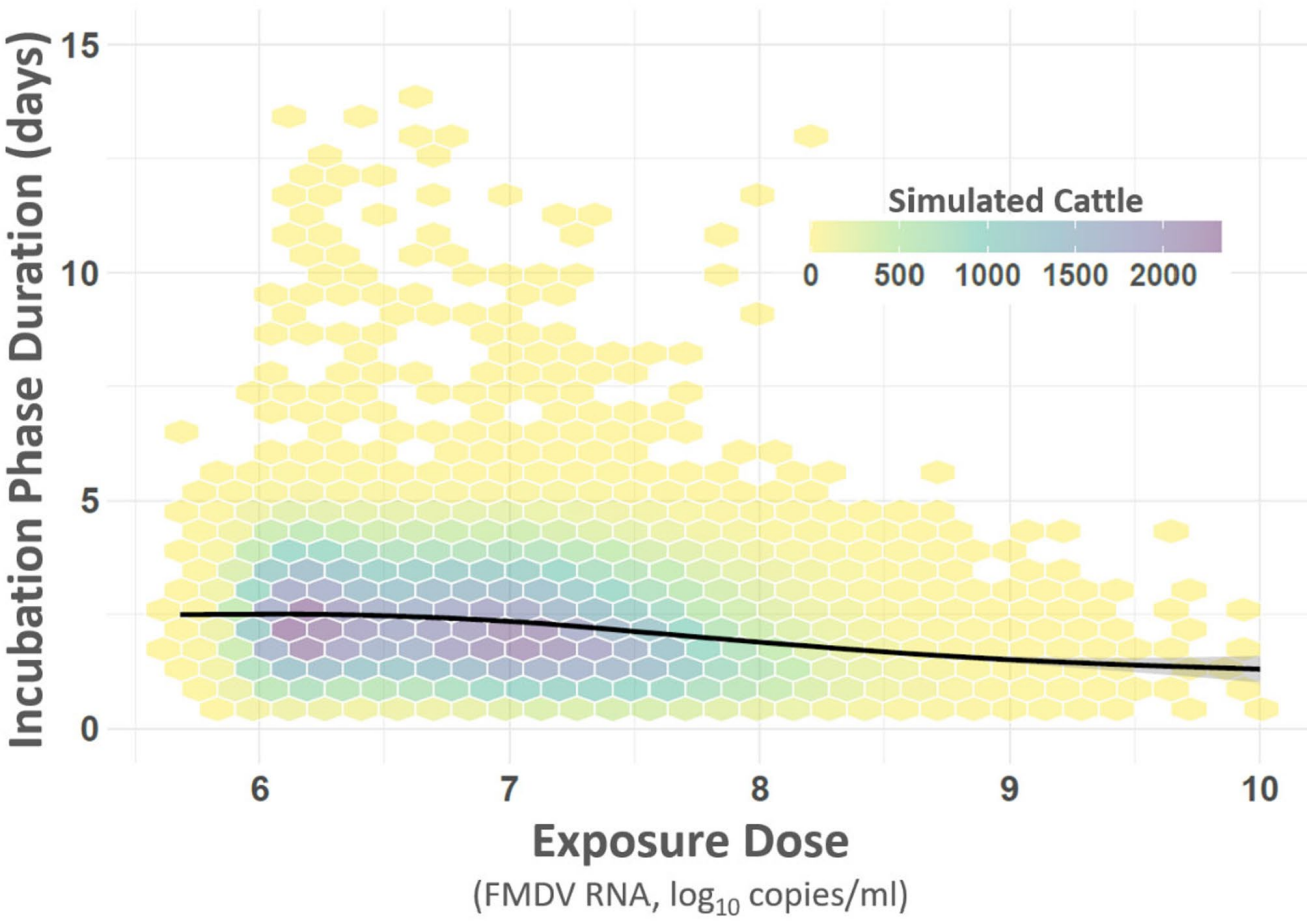
Contact group dose–response relationship. Figure displays the relationship between FMDV virus quantities within the infecting animals and resulting change in incubation phase duration experienced by infected animals. Vertical axis at left shows duration of the incubation phase (days), horizontal axis indicates virus quantity. Plot area summarizes results for 180,000 simulated animals. Due to the high number of simulated animals, data has been binned to hexagons and color-coded according to inset legend at top right to indicate data distribution. Smooth line shows the statistically significant, non-linear decrease in incubation period duration with increased virus load contribution by contacting animal.

The relationship between the effective viral exposure dose and the incubation period was evaluated using a generalized additive model (GAM) and a linear regression model (Fig. 7). The GAM identified a significant non-linear relationship between dose and incubation period, as evidenced by the smooth term (edf = 5.76, F = 891.4, p < 2 × 10⁻^1^⁶), with an adjusted R^2^ of 0.038, indicating modest explanatory power. The linear model also showed a significant inverse relationship between dose and incubation period (β = –4.74, t = –75.75, p < 2 × 10⁻^1^⁶), with an adjusted R^2^ of 0.035. This model estimated that the incubation period shortened by approximately 4.74 days for each tenfold (1 log₁₀) increase in viral dose, consistent with a negative dose–response relationship.

However, comparison of model fit using Akaike Information Criterion (AIC) strongly favored the GAM (AIC = 1,435,265) over the linear model (AIC = 1,435,712), indicating substantial improvement in fit with the inclusion of non-linearity (ΔAIC = 447). The GAM curve suggested a diminishing effect of dose at higher exposure levels, consistent with biological saturation, and a potential threshold effect at lower doses. (Fig. 7).

#### Simulated within-host infection dynamics

**Fig. 8:**
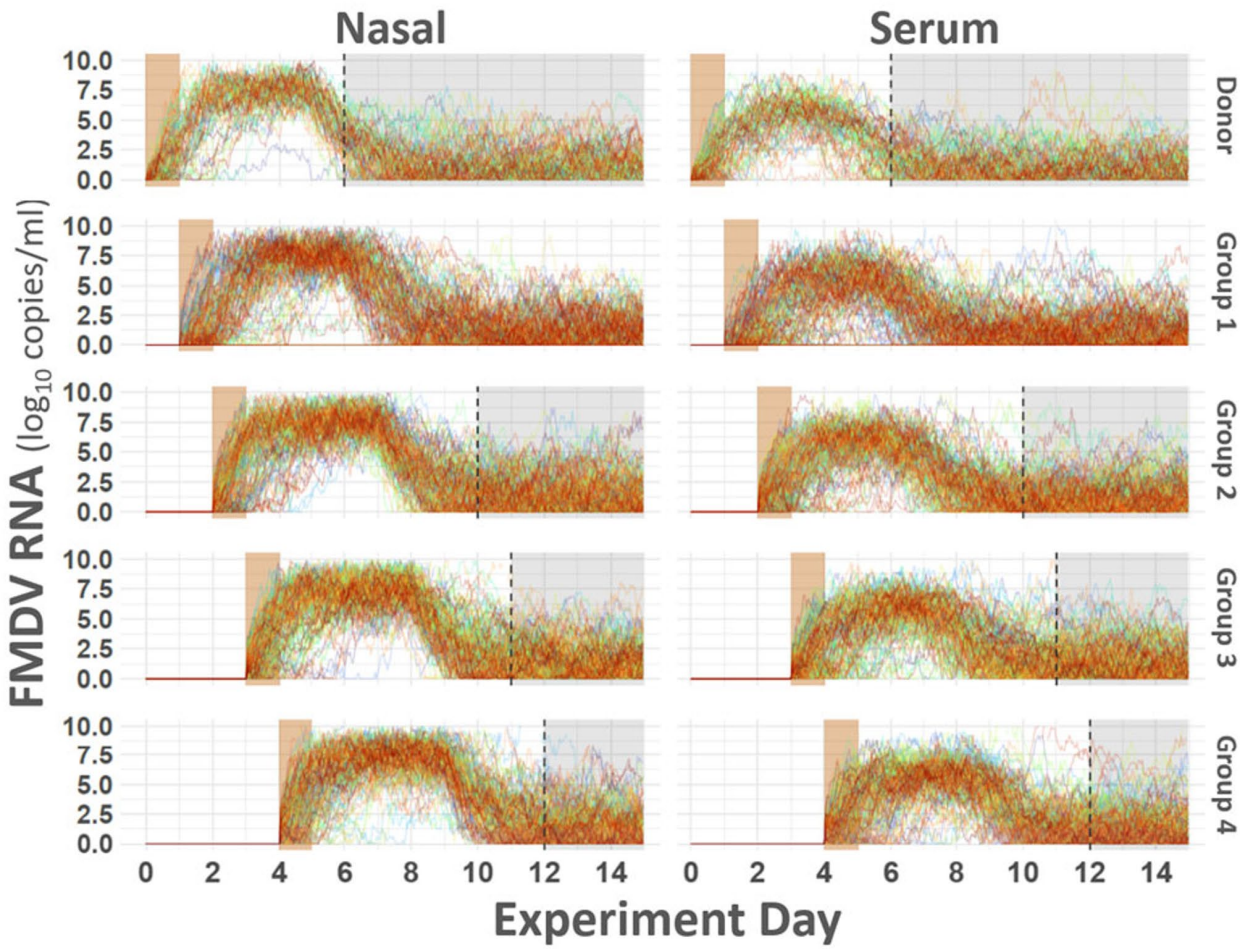
Simulated within-host infection dynamics. Figure illustrates trajectories of FMDV RNA detection in nasal swabs and serum resulting from room-to-room simulation. Vertical axes at left describe FMDV RNA quantity, horizontal axes at bottom correspond to experiment day. Panel rows are organized by experiment group as labeled along right side margin. Columns are partitioned to show estimates for nasal shedding (left) and serum (right). Plotted smooth lines represent individual animals and are arbitrarily colored. Brown shaded regions near start of increase highlight exposure period to donors. Vertical dashed lines within plot area demarcate the end of each group’s empirically observed period. Shaded gray regions right of dashed lines indicate periods simulated but not observed during the experiment. Note that several trajectories are parallel and trace the horizontal axis in Group 1, indicating non-infection in Group 1 (similar to what was observed in the empirical animal trial). The figure displays simulated trajectories produced by 40 randomly selected trials (720 animals) intended for demonstrative purposes only.

Simulated within-host viral trajectories for nasal shedding and serum FMDV RNA levels mirrored empirical patterns observed in the room-to-room experiment (Fig. 8). Group 1 had lower initial virus shedding than later contact groups, consistent with a low infection rate due to early-stage donor shedding. In contrast, later groups had steeper and earlier increases in viral RNA quantities, reflecting higher donor viral loads at exposure time. It is worth noting that only approximately 1 in 10 simulations resulted in no Group 1 infections, matching the Group 1 empirical outcome. (Fig. 8).

#### Within-herd outbreak simulations

Outcomes from within-herd outbreak simulations demonstrated marked differences between scenarios with and without inclusion of preclinical transmission (Table 1). There was no statistical difference in the timing of occurrence of the first clinical case of FMD between the two scenarios (including preclinical transmission, median: 2.81 days, 95% CI 2.33–3.51; clinical-only scenario, median: 2.62 days, 95% CI 2.06–3.22). However, at the time of first clinical detection, the number of infectious animals was substantially higher when preclinical transmission was included (median: 5.02 animals, range 3.00–9.27) compared to the clinical only scenario (median: 1.00), where only the first animal with clinical signs was infectious.

**Table 1:** Within-herd outbreak simulation summary.

Statistic	Preclinical and clinical	Clinical only
First clinical case time (days)	2.81 (2.33, 3.51)	2.62 (2.06, 3.22)
Total infectious at first clinical Case	5.02 (3.00, 9.27)	1.00 (1.00, 1.00)
peak prevalence time (days)	8.36 (7.65, 9.13)	12.14 (11.08, 13.42)
Generation time (days)	1.84 (1.34, 2.51)	3.06 (2.23, 3.92)
Reproduction number (R_0_)	10.0 (3.0, 17.0)	9.0 (2.0, 18.0)

Summary statistics from herd outbreak simulations for the combined preclinical- and clinical, and clinical-only scenarios. Values preceding parentheses are the median, parenthetical values represent the 95th percentile. Each scenario was subject to 1000 independent trials with a 100-animal herd size (100,000 animals per scenario). Note that associated prevalence curves for each scenario are provided in Fig. 10.

Peak prevalence, the time with the highest number of concurrently infectious animals, occurred earlier in the scenario including preclinical transmission (median: 8.36 days, range 7.65–9.13) relative to the clinical-only scenario (median: 12.14 days, range 11.08–13.42), highlighting faster within-herd spread when preclinical animals contributed to transmission (Table 1). Similarly, generation times were shorter with preclinical transmission included (median: 1.84 days, range 1.34–2.51) compared to the clinical-only scenario (median: 3.06 days, range 2.23–3.92), further reflecting faster transmission dynamics.

**Fig. 9:**
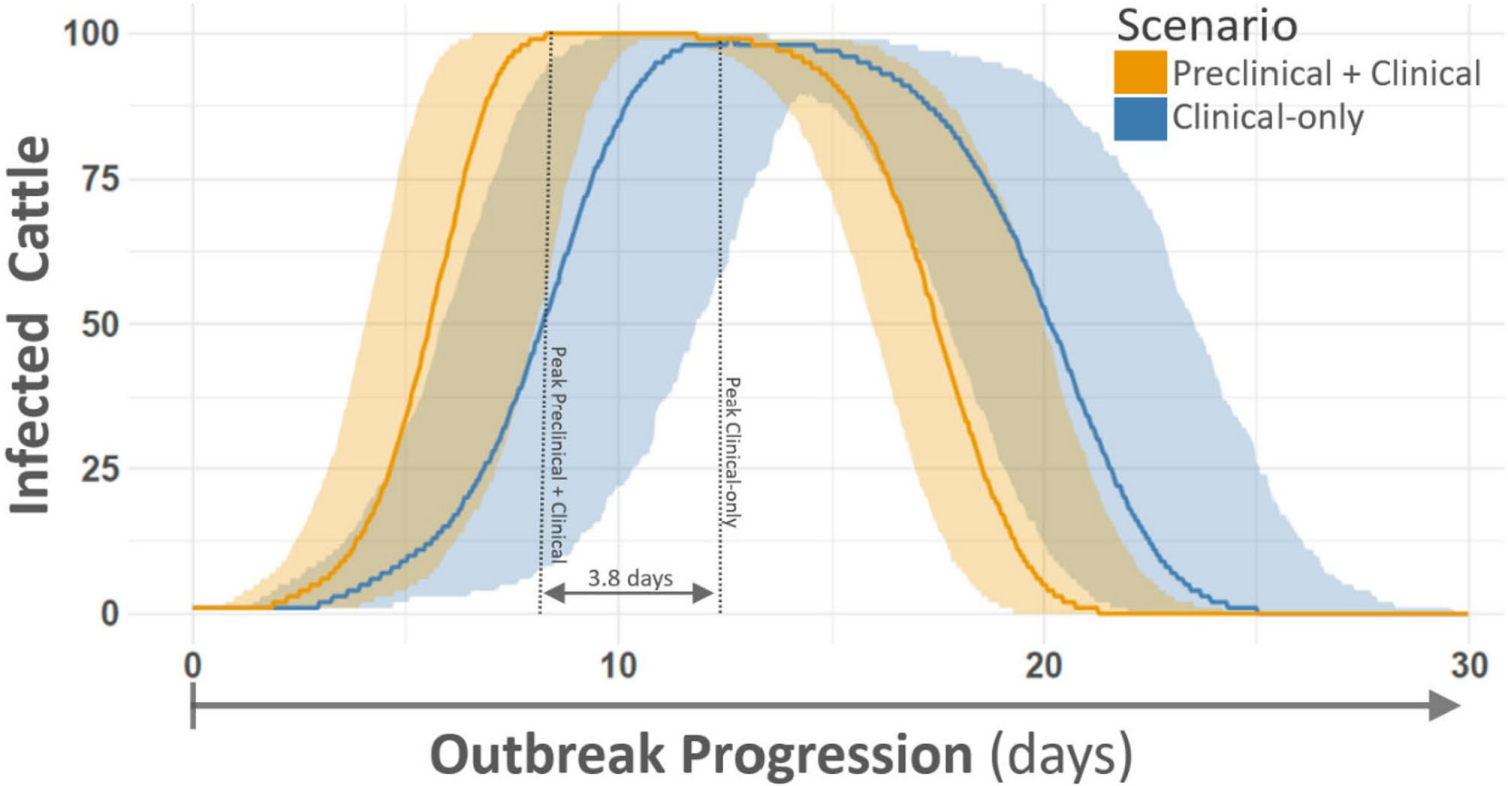
Within-herd outbreak simulation infection prevalence curves. Figure depicts FMDV infections (prevalence) from herd outbreak simulations for scenarios with and without preclinical transmission. Vertical axis at left describes the number of infected cattle, horizontal axis gives outbreak progression in hours. Smooth lines represent median estimates with surrounding ribbons showing the 25th-75th interquartile range. Lines and ribbons are color-coded according to inset legend to distinguish the combined preclinical and clinical transmission scenario from that with transmission restricted to clinical animals only. Vertical dotted lines within plot area demarcate peak prevalence times. Each scenario was subject to 1000 independent trials with a 100-animal herd size (100,000 animals per scenario). Note that the peak prevalence of the scenario that included preclinical transmission occurred several days before peak prevalence in the clinical-only scenario, indicating faster rates of spread within herds.

The estimated basic reproduction number (R_0_) was similar between scenarios, with medians of 10.0 (range 3.0–17.0) and 9.0 (range 2.0–18.0) in the combined preclinical and clinical, and clinical-only scenarios, respectively. This suggests that while overall transmission potential remained comparable, preclinical transmission conditions led to earlier and faster spread due to a larger number of infectious animals before clinical onset (Fig. 9).

**Fig. 10:**
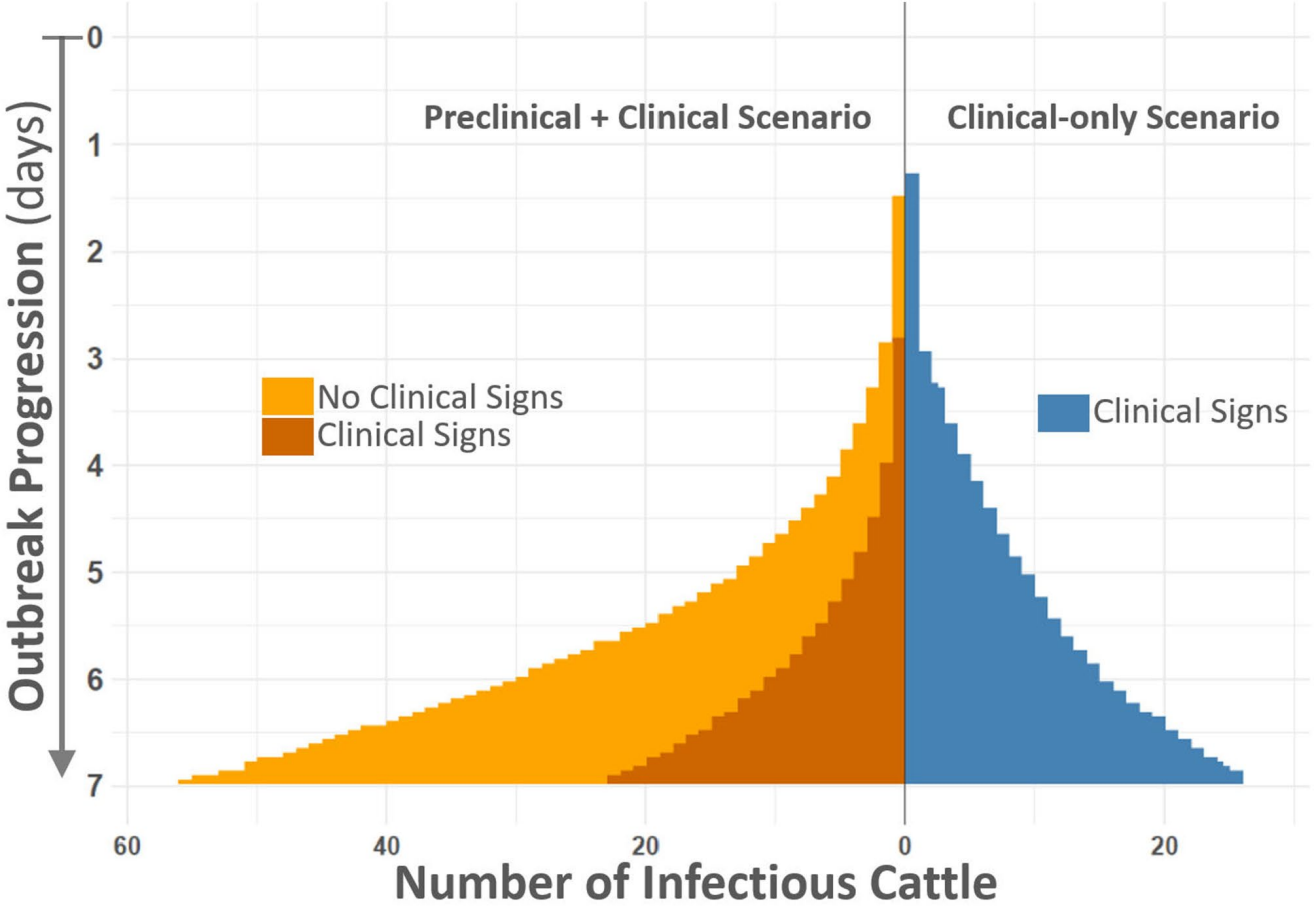
Cumulative infectious animals at one week. Figure compares median accumulation of infectious animals between simulated scenarios with and without preclinical infectiousness. Vertical axis displays outbreak progression from time of initial infection (top of figure, 1 animal, time 0) through the first week of outbreak (bottom of figure, day 7). Horizontal axis describes the number of infectious animals. Results for the combined preclinical- and clinical, and clinical-only scenarios are distinguished by color according to inset keys, with the combined preclinical- and clinical scenario further subdivided to differentiate between infectious animals with and without clinical signs. Each scenario was subject to 1000 independent trials with a 100-animal herd size (100,000 animals per scenario). Note that by day 7, approximately 53 infectious animals are contributing to disease transmission in the combined preclinical-and clinical scenario, whereas about 27 are actively transmitting in the clinical-only scenario.

**Fig. 11:**
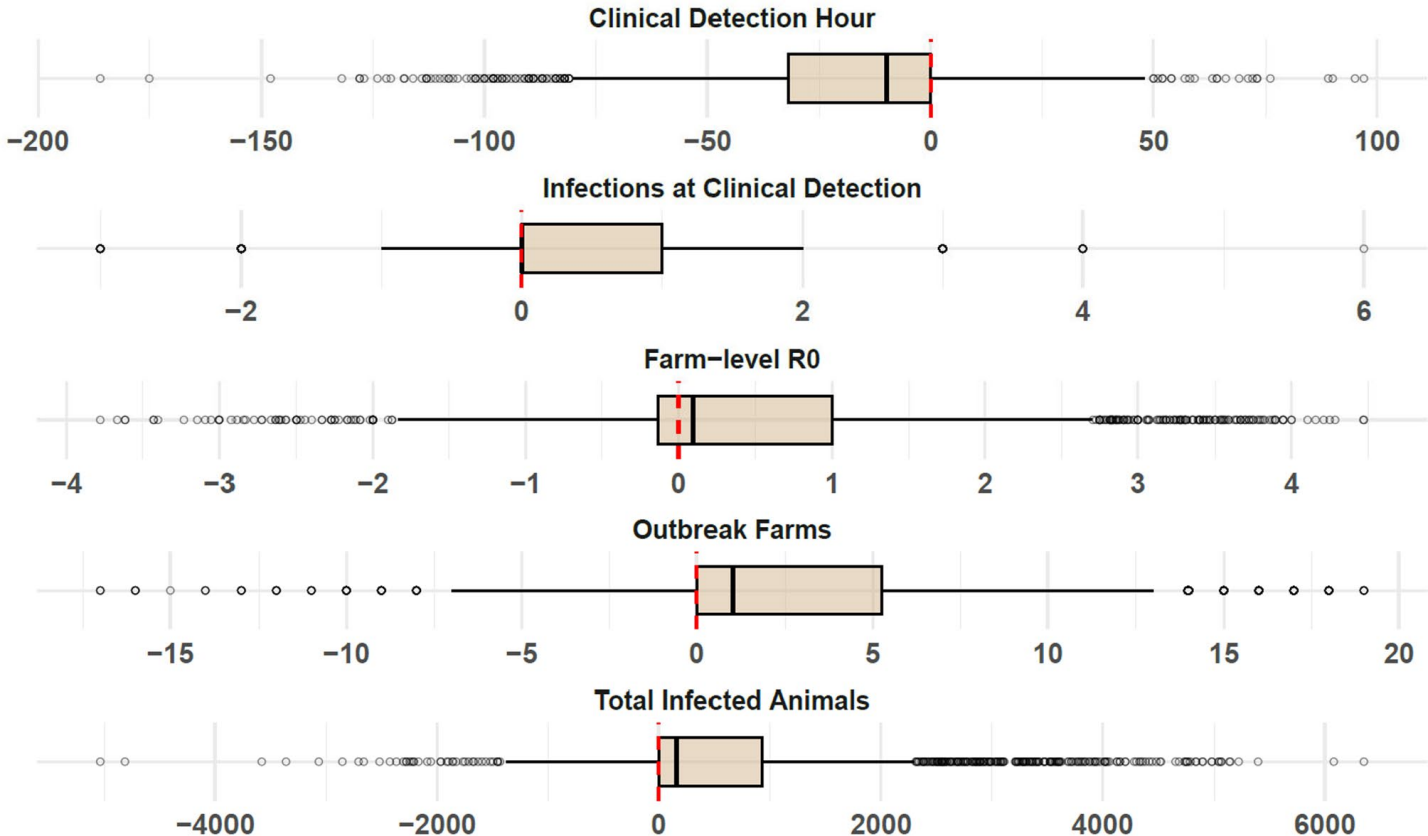
Distribution of within-iteration differences in simulated between-farm outbreak metrics. Each box represents the difference between scenarios with and without preclinical infectiousness. Departures from zero on horizontal axes (vertical red line) indicate influence of preclinical infectiousness. Measures show farm-level reproduction number (R₀), number of infected farms, number of infections at the time of clinical detection, and total infected animals increase with preclinical infectiousness. Clinical detection hour indicates that outbreaks occur more rapidly in scenarios including preclinical infectiousness.

Herd-level prevalence curves (Fig. 10) further illustrate the rapid spread in the scenario that included preclinical transmission, with peak prevalence occurring several days earlier than in the clinical-only scenario. The cumulative number of infectious animals at one week (Fig. 11) highlights the magnitude of differences between scenarios, with approximately 53 animals contributing to transmission in the combined preclinical and clinical scenario compared to 27 animals in the clinical-only scenario. These results underscore how even modest preclinical shedding can substantially increase early outbreak size and reduce the window for effective intervention.

#### Between-farm outbreak simulations

**Fig. 12:**
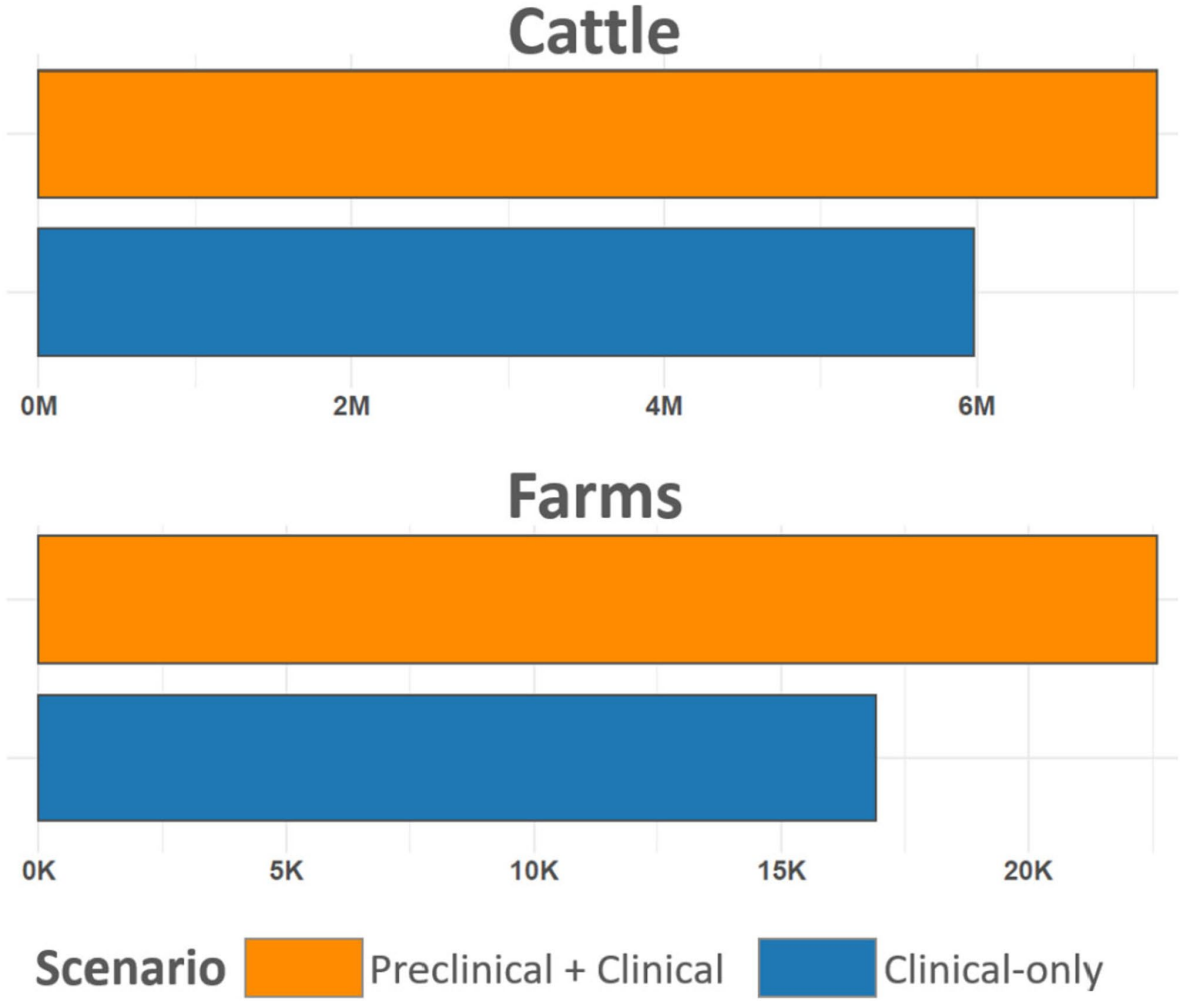
Comparison of total infected cattle and farms in between-farm simulation. Figure depicts the total number of cattle (top, millions) and farms (bottom, thousands) across all simulated farm-to-farm outbreaks. Bars are color coded according to legend at bottom to distinguish scenarios with and without preclinical infectiousness.

Simulated between-farm outbreaks revealed consistent and substantial differences between scenarios with and without preclinical infectiousness. Using paired simulations that held structural and stochastic parameters constant except for including preclinical transmission, increases in outbreak magnitude, velocity, and spread resulted under the preclinical scenario (Figs. 11 and 12). Analysis of differences between paired scenarios demonstrated that including preclinical transmission led to increased farm-level reproduction number (R₀), total number of infected farms, number of infected animals at the time of first clinical detection, and total number of infected animals throughout each outbreak. These differences were consistently positive across nearly all simulation pairs. However, the timing of clinical detection occurred earlier in the preclinical scenario (Fig. 11).

Cumulative outcome comparisons further reinforced these effects. When aggregating across all 2000 outbreak pairs, the preclinical scenario resulted in approximately 7.15 million infected animals across 22,595 farms, compared to 5.98 million animals across 16,903 farms in the clinical-only scenario (Fig. 12). These differences represent a 19.6% increase in infected animals and a 33.7% increase in the number of affected farms attributable solely to the inclusion of preclinical infectiousness. All pairwise comparisons were statistically significant based on Wilcoxon signed-rank tests (p < 10⁻^12^ for all outcomes), demonstrating that the observed differences were not due to stochastic variation alone.

Together, these results emphasize that even modest transmission levels during the preclinical phase can substantially accelerate and expand outbreak scale at the between-farm level. While total transmission potential (R₀) remains within a comparable range, the contribution of preclinical infectiousness to earlier, undetectable transmission stages reduces the effectiveness of detection-based passive surveillance, increasing outbreak impact and compressing response timelines.

## Discussion

Preclinical transmission is a critical but under-characterized feature of FMDV epidemiology. This study provides direct experimental evidence that infected cattle can transmit FMDV during the incubation period, before the appearance of clinical signs, and demonstrates through calibrated simulations that even a modest number of preclinical infectious animals can substantially accelerate transmission dynamics both within herds and across larger farm networks. These findings emphasize the role of early, undetected transmission in shaping outbreak trajectories and highlight the limitations of control strategies that rely solely on the detection of clinical disease.

In the empirical component of our study, two donor cattle infected with FMDV A24 Cruzeiro were sequentially co-mingled with four groups of susceptible cattle, each housed in a separate isolation room, for consecutive 24-h periods. This design approximated natural exposure conditions by allowing the donors to interact with different groups of susceptible animals over time. The outcomes of this experimental transmission study demonstrated that infected cattle could transmit FMDV at least 24 h before the appearance of vesicular lesions, highlighting the potential for preclinical transmission. Subsequent groups of exposed animals had progressively more rapid disease progression, correlated with the appearance and advancement of clinical disease in the donor animals during the contact exposures. The observed earlier onset of clinical disease and greater severity of early infection in the later contact groups suggests that these groups were subjected to a greater cumulative virus exposure during their 24 h exposure period.

The finding of a more gradual onset of infection in animals exposed to the infected donor animals during the early infectious period (corresponding to a lower cumulative virus exposure) is consistent with previous findings from a FMDV transmission study in pigs^19^. In both cases, the exposed animals eventually reached full lesion scores, corresponding to fully disseminated infection, but the progression was slower and less dramatic compared to animals that had been exposed to the donors during more advanced stages of infection. Interestingly, the FMDV infection dynamics in the two donor cattle that had been infected through intra-nasopharyngeal deposition of virus inoculum were more similar to the infection dynamics observed in the earliest of the infected contact groups (group 2) than to those seen in the two later exposed groups. This suggests that the approach of intra-nasopharyngeal deposition of the virus inoculum that was used for the donors (10^6^TCID_50_) more closely resembled the pre-clinical exposure of contact group 2, than the clinical contact exposure of the later groups. Combined, these findings suggest a direct relationship between the cumulative virus exposure and the duration of distinct phases of infection in FMDV-infected cattle, which is also consistent with a previous investigation based on modeling of FMDV infection phases in pigs^20^. Such variation in disease progression would likely be more pronounced under field conditions when the magnitude and duration of virus exposure can be expected to be more variable.

The cumulative virus exposure during contact transmission trials is a product of the quantity of infectious virus shed by donor animals, as well as the intensity and duration of animal contacts. Transmission occurred rapidly after 72 hpi, when donor animals were in the early stages of clinical FMD. It is reasonable to assume that more extended exposure periods are required to increase the transmission probability when donors are in the preclinical infectious phase and shedding lower quantities of virus. In this study, infected donor animals were moved into new isolation rooms for each contact exposure period, preventing environmental accumulation of contagion that could otherwise contribute to transmission. Due to logistical constraints, measurements of virus shedding and observation of clinical signs in individual animals were only obtained at 24 h intervals. Additionally, it was not possible to quantitate the characteristics or intensity of animal interactions during the exposure periods. These factors limit the appropriateness of extrapolating a precise value of virus shedding associated with the onset of infectiousness in the donor animals.

Differences in effective exposure dose and strain-specific FMDV variability may explain dissimilar outcomes reported in studies investigating preclinical FMDV transmission in cattle. For instance, Charleston et al.^13^ evaluated donor animals’ ability to transmit infection for only 8-h periods, once every other day, concluding that FMDV was transmissible for a shorter duration than previously presumed and that preclinical transmission did not occur. The current study suggests that longer exposure durations, as would typically occur with group-housed animals, increase the probability of transmission, particularly during the preclinical infectious phase when virus shedding is moderate.

Agent-based modeling (ABM) provided mechanistic insight into these observations and allowed extension to hypothetical and scaled-up outbreak scenarios. Within-host simulations accurately reproduced empirical viral trajectories and infection phase durations, validating the model’s capacity to capture exposure-dependent infection kinetics. There was some variation between empirical and simulated scenarios related to infectiousness during the 24–48 hpi time frame. While there was no transmission observed during this time period in the empirical study, the model predicted that transmission would occur in nine of ten simulations. This discrepancy can likely be explained by constraints related to conducting experimental studies in large animals in high containment laboratory facilities. While the ABM assumes a continuous increase in infectiousness over time, the empirical study allowed only coarse periodic measurements of virus shedding and limited the number of experimental iterations (exposure periods) that could be assessed. Additionally, while the ABM has the clear advantage of allowing for repeated simulations, it was not feasible to perform more than one replication of the animal experiment.

Herd-level simulations demonstrated that including preclinical transmission led to shorter generation times, earlier prevalence peaks, and substantially more infectious animals present before the appearance of clinical signs. These dynamics are especially relevant to real-world disease spread, where undetected but subclinically infectious animals may be moved between herds, facilitating silent dissemination.

The within-herd basic reproduction number (R0) from the simulations was consistent across combined preclinical and clinical, and clinical-only transmission scenarios, with median values of 10.0 (3.0, 17.0) and 9.0 (2.0, 18.0), respectively. These values align with previous estimates from experimental and modeling studies^21,22^, where R0 values ranged from 2.9 to 21.9 using nasal fluid viral growth as an infectiousness measure. These findings suggest that while the overall potential for transmission is comparable, preclinical transmission dramatically alters the temporal dynamics of outbreaks, leading to faster dissemination of disease within herds.

Extending beyond individual herds, the simulation of between-farm transmission dynamics using matched scenarios with and without preclinical infectiousness revealed that even modest preclinical shedding can substantially increase outbreak scale and speed at larger geographic scales. Outbreaks incorporating preclinical transmission resulted in a 19.6% increase in the total number of infected animals and a 33.7% increase in the number of affected farms, compared to clinical-only scenarios. These differences were consistent across all simulated outbreaks and were statistically significant in all comparisons. Importantly, preclinical transmission also led to earlier outbreaks, reducing the effectiveness of passive surveillance. These results demonstrate that preclinical infectiousness, despite not altering the overall transmission potential between farms, significantly amplifies outbreak velocity and geographic spread, thereby challenging control at the regional scale.

This extent of preclinical shedding points to the challenges in managing FMDV outbreaks through reactive control measures, such as case isolation or culling. The effectiveness of reactive measures is reduced when a substantial proportion of transmission occurs before clinical signs are detected. The simulation results presented here reinforce this concern, as the number of simulations with θ proportions above the median suggests that preclinical transmission could enable outbreaks to progress significantly before detection.

The asymptomatic nature of preclinical infections poses significant challenges for disease detection and control. Without visible clinical signs, preclinical infections will likely go unnoticed within herds, allowing transmission to continue unchecked. This was evident in the simulations, where more animals were infectious during the early stages of outbreaks in the combined preclinical and clinical scenario, increasing the likelihood of rapid and widespread dissemination. In field settings, the inability to detect preclinical infections can lead to delays in implementing control measures, amplifying the scope and severity of outbreaks. The risk is further exacerbated by the movement of cattle between locations. Striving to detect FMDV incursions prior to the appearance of clinical signs would likely not be feasible in non-endemic regions due to the excessive resources that would be required. However, less intensive approaches, such as testing of bulk-tank milk^23,24^ , air sampling^25,26^, or routine sampling of a subset of animals on each farm^21^, could potentially aid in early detection of infection in known high-risk areas.

In absence of detection of infection, subclinically infected animals transported to different farms, markets, or facilities can unknowingly seed new outbreaks before clinical symptoms emerge. Simulations highlighted how accelerated timelines in scenarios with preclinical transmission could lead to secondary outbreaks that are already well-established by the time clinical signs are observed. This aligns with historical accounts of FMDV outbreaks, such as the 2001 epidemic in the U.K., where asymptomatic sheep likely contributed to early and widespread dissemination^16,27^.

Moreover, the enhanced transmission rates associated with preclinical infectiousness mean that control measures based solely on detecting clinical cases may be insufficient. Even if clinical cases are identified and culled, simulations suggest that a substantial portion of the herd could already be infected and infectious. This concept is consistent with reports of FMD outbreaks as well as simulation studies, which have suggested that culling and isolation may not be sufficient to control FMD outbreaks in areas of high livestock density^28–31^. The simulated scenarios in the current study did not involve movement restrictions on farms other than those directly affected. In non-endemic regions, movement restrictions applied upon detection of FMD would typically affect all animal holdings within a certain radius from the affected premises, potentially limiting regional transmission once the first case had been detected.

Generalized additive model (GAM) analysis provided additional evidence for a dose–response relationship, revealing that higher viral doses were associated with shorter incubation periods. The non-linear relationship observed suggests that while higher doses accelerate disease progression, the effect diminishes at the highest doses, potentially reflecting biological saturation effects. This nuanced understanding of dose effects on FMDV pathogenesis highlights the need for further investigation to refine models and improve predictive accuracy^32,33^.

As with all investigations involving housing livestock within high-containment laboratory facilities, there are clear limitations in the current study. FMDV exists as seven serotypes with multitudes of distinct strains within each serotype, each of which possesses some unique characteristics. If further considering the wide host range, as well as known and unknown differences in susceptibility between different breeds and age categories of host animals, the potential experimental design options become indefinite. The observations from the empirical part of the current investigation can naturally not be representative of all possible virus-host combinations. However, combining the empirical observations with simulated scenarios provides the ability of assessing how the observed transmission dynamics could affect a larger-scale disease outbreak.

The empirical, analytical, and simulation results emphasize the critical role of viral exposure conditions in FMDV transmission and progression. These findings highlight the complexity of dose–response relationships, the importance of considering preclinical transmission in disease dynamics, and the value of integrated methodologies for informing effective control strategies. In field settings, variability in exposure conditions and the presence of subclinically infected animals could contribute to rapid and widespread dissemination, emphasizing the need for targeted interventions that account for these dynamics.

## Methods

### Empirical experimental study

#### Virus and animals

The virus used in this current investigation was a bovine-derived isolate of FMDV strain A24 Cruzeiro. The pathogenesis of this virus in cattle has been characterized in detail in previous publications^11,12,34^. The animals used were Holstein heifers of approximately 400 lb procured from a USDA-certified vendor. The animal experiment was carried out in the BSL3-Ag facility at the Plum Island Animal Disease Center (PIADC), New York. The study protocols were approved by the Plum Island Animal Disease Center Institutional Animal Care and Use Committee which functions to ensure responsible and humane treatment of experimental animals (protocol 209.02-21R). All procedures were carried out in accordance with relevant institutional regulations and guidelines. The outcome of the experiment are reported in accordance with ARRIVE guidelines.

#### Study design

The described experiment included 18 cattle. Two individuals were randomly selected as “donors” and were infected by intra-nasopharyngeal (INP) deposition of 10^6^ TCID_50_ FMDV A24 on study day 0. At 24 h post inoculation (hpi) the two donors were relocated to a different isolation room that housed four recipient cattle (contact group 1). After 24 h of co-habitation with contact group 1, at 48 hpi of the donors, the two donors were again relocated to a different room housing four new cattle (contact group 2). This was continued until four contact groups had been co-housed with the donors for 24 h each (Fig. 1). At that time, which corresponded to 120hpi, the two donors were removed from the study and humanely euthanized. The contact-exposed animals were monitored for up to 14 days after exposure of the donors. However, the experiment was terminated at 7 days post-exposure (dpe) for animal welfare reasons, for groups in which the majority of animals had developed clinical FMD. Nasal swabs and blood samples were collected at 24 h intervals through the first 5 days of observation of each group and then again at days 7, 10, and 14 dpe. Oropharyngeal fluid (OPF) was collected through probang sampling on days 10 and 14 for the animals that remained in the experiment. Whole blood in serum-separation tubes and nasal tampons were centrifuged for extraction of serum and nasal fluid immediately after sample collection, and samples were stored at − 70 °C until further processing. OPF samples were homogenized using a 16G 6″ steel cannula attached to a syringe. One aliquot of OPF designated for virus isolation (VI) was treated with 1,1,2- trichlorotrifluoroethane (TTE) before freezing.

Clinical examination under sedation (intra-muscular injection of Xylazine hydrochloride (0.66 mg/kg), reversed by intra-venous injection of Tolazoline (2.0 mg/kg)) to enable thorough examination of the feet was done at 24 h intervals for the donor cattle (corresponding to the beginning and end of each contact-exposure period) and approximately every other day for the contact-exposed cattle. Clinical examination without sedation, focused on lesion detection on the muzzle, in the oral cavities, and on the coronary bands, as well as monitoring of rectal temperatures, were done daily for all animals. The progression of clinical infection (lesion distribution) was measured by a quantitative cumulative scoring system. In brief, any vesicular lesions observed within the oral cavity (dental pad, tongue, gingiva), lips, or nostrils counted for 1 point, with additional points added for vesicles on any of four feet (1 point per foot), resulting in a maximum score of 5. Animals were euthanized under sedation (Xylazine 0.66 mg/kg IM) by intravenous injection of pentobarbital (90 mg/kg).

#### FMDV RNA detection

Serum, nasal fluid and OPF samples were analyzed using quantitative real-time RT-PCR (qRT-PCR), targeting the 3D region of the FMDV genome^35^ with forward and reverse primers adapted from Rasmussen et al.^36^, and chemistry and cycling conditions as previously described^37^.

#### Virus isolation

Aliquots of TTE-treated OPF samples obtained from group 1 animals on 10 and 14 dpe were cleared of debris and potential bacterial contamination by centrifugation through Spin-X® filter columns (pore size 0.45 μm, Sigma-Aldrich). Virus isolation (VI) was performed using LFBK-αvβ6 cells^38,39^, following a protocol previously described^40^. Absence of amplified FMDV in VI supernatants was confirmed by qRT-PCR.

#### FMDV serology

Serum samples obtained on the first and last study days for animals in contact group 1 were analyzed for the presence of neutralizing antibodies against FMDV A24 using the methodology described in detail previously^41^.

### Statistical analysis and mathematical modeling

#### Statistics evaluation

The study employed four complementary analytical approaches to address distinct aspects of the data collected from the animal experiment: (1) repeated measures ANOVAs to evaluate time-varying differences in nasal viral load, serum viral load, and lesion scores among experimental groups; (2) survival analysis to model disease phase durations, including latent, preclinical, and incubation phases; (3) generalized additive models to assess relationships between incubation period duration and virus dose; and (4) an agent-based model (ABM) to simulate within-host viral dynamics, between-host virus transmission within a herd, and between-farm spread. Each approach is described below.

Time-varying differences in nasal viral load, serum viral load, and lesion scores among experimental groups were assessed using repeated measures ANOVAs via the ezANOVA function from the *ez* R package^42^. The analyses included both within-subject factors (days post-exposure) and between-subject factors (donor and contact groups), with the dependent variables as nasal viral load, serum viral load, and lesion scores. Mauchly’s test was used to evaluate the assumption of sphericity, and when violations were detected, Greenhouse–Geisser and Huynh–Feldt corrections were applied.

Disease phase durations were modeled using survival analysis, which was implemented using the INLA R-package^43,44^. Latent and incubation phase durations were modeled as time-to-event outcomes with an exponential survival distribution. The preclinical infectious phase duration was approximated by subtracting the latent phase duration from that of the incubation. To incorporate time-varying viral shedding dynamics, a first-order random walk was included as a random effect to model the interaction between time and viral shedding. This approach provided a flexible, non-linear representation of virus shedding over phase durations. A penalized complexity (PC) prior was applied to random walk precision, ensuring regularization while penalizing deviations from parsimony.

To evaluate the presence and linearity of incubation period dose effects in simulated results, we compared two statistical models: a generalized additive model (GAM) and a linear regression model. The incubation period was the dependent variable, and the viral dose received at the time of infection was the independent variable. The GAM model included a smoothing function for non-linear relationships, while the linear model assumed a direct linear relationship between dose and incubation period. Both models were fit to the data using the mgcv R package (Wood 2011).

Model fit was assessed and compared using Akaike Information Criterion (AIC) values. AIC was used to determine whether the non-linear GAM model provided a better fit to the data than the simpler linear model. This analysis enabled us to test for the significance of the dose–effect and assess whether the relationship between dose and incubation period was linear or non-linear.

#### Agent-based models

Agent-based models (ABMs) are computational frameworks that simulate the behavior and interactions of autonomous agents to assess their cumulative effects on the system. The ABM developed for this study comprises three integrated modules, room-to-room, within-herd, and between-farm, each designed to represent different epidemiological scales of FMDV transmission among cattle. The following description provides a conceptual overview of the ABM structure and logic. Full implementation details, including the source code, parameter definitions, and configuration files, are provided in the associated online material (https://osf.io/qf2wr/) following the Overview, Design concepts and Details (ODD) protocol^45^.

At the core of the model, individual agents represent bovine hosts that undergo within-host viral dynamics and participate in between-agent transmission events. To capture biological variability among individuals, agents are initialized with parameter values drawn from empirically grounded statistical distributions, reflecting differences in viral growth rates, immune responses, and thresholds for infectiousness and clinical onset. By assigning individualized parameters, the model simulated a heterogeneous population in which each agent’s disease progression could vary, closely mirroring real-world scenarios.

*Within-host viral dynamics* The viral dynamics of each agent were modeled using logistic growth equations that captured the replication and clearance of the virus within the host. Mathematically, the change in viral load **V**_**(t)**_ over time **t** was described by:

$$\frac{dV}{{dt}} = rV\left( {1 - \frac{V}{K}} \right) - cV + \epsilon$$

where:

- ***r*** was the intrinsic growth rate of the virus, unique to each agent.

- ***K*** was the carrying capacity, representing the maximum viral load the host could sustain.

- **c** was the clearance rate due to the host’s immune response.

- $\epsilon$ was a stochastic noise term accounting for random biological fluctuations.

This equation reflected the biological process wherein the virus initially underwent exponential growth. It slowed as it approached the carrying capacity due to resource limitations and immune responses and might exhibit temporary plateaus or fluctuations around K . The decline phase represented the immune system effectively reducing the viral load through a combination of innate and acquired immunity.

Having identified possible dose–response effects during the animal experiment, agent-to-agent transmission was modeled using a modified Hill equation to describe the dose–response relationship and calculate effective dose efficiency, which influenced the initial viral load in a newly infected agent. The effective dose efficiency was given by:

$${\mathrm{Dose}}_{{{\mathrm{Eff}}}} = \frac{{E_{{{\mathrm{max}}}} }}{{1 + {\mathrm{exp}}\left( { - \lambda \left( {\frac{{V_{{{\mathrm{inf}}}} }}{{K_{{{\mathrm{inf}}}} }} - \theta_{{{\mathrm{adj}}}} } \right)} \right)}}$$

where:

- ***E***_***max***_ was the maximum dose efficiency, representing the upper limit of efficiency with which the viral dose contributes to infection.

- ${\boldsymbol{\lambda}}$ was the dose scaling factor, influencing the steepness of the dose–response curve.

- $\frac{{{\boldsymbol{V}}_{{{\mathrm{inf}}}} }}{{{\boldsymbol{K}}_{{{\mathrm{inf}}}} }}$ was the viral load ratio of the infectious agent.

- ${\boldsymbol{\theta}}_{{{\mathrm{adj}}}}$ was the adjusted dose threshold, calibrated to ensure that the dose efficiency reached a specified value at a particular viral load ratio.

Once the effective dose efficiency was calculated, the initial viral load in the newly infected agent was determined by:

$$V_{{{\mathrm{initial}}}} = {\mathrm{Dose}}_{{{\mathrm{Eff}}}} \times D$$

where **D** was the viral dose received by the susceptible agent.

*Room-to-room module *This module emulates the experimental design used in the accompanying empirical study, in which donor cattle sequentially enter rooms containing naive recipients. Donor agents are initialized as infected and move between rooms at predefined intervals, exposing new groups of susceptible agents. Transmission is localized within each room and depends on the average nasal viral load of infectious agents. Within-host viral dynamics continue for each agent over time, and symptoms emerge once both nasal and serum viral loads surpass respective clinical thresholds.

*Within-herd module* To generalize transmission dynamics to population scales, a within-herd module was developed. This module retains the individualized viral dynamics and dose–response transmission logic from the room-to-room framework but assumes homogeneous mixing. Agents interact via stochastic pairwise contacts governed by a per-hour contact rate of 0.15. Transmission events occur based on calculated dose efficiency, and resulting infections proceed according to individualized within-host trajectories. Infectiousness is linked to either preclinical or clinical viral loads, depending on the model setting, controlling whether transmission is permitted before symptom onset. Recovery is modeled as a function of individual-specific recovery times, after which agents no longer contribute to transmission.

*Between-farm module* To simulate regional-scale transmission, the ABM incorporates a network-based between-farm module. Each node in the network represents a cattle farm populated by agents initialized and simulated using the within-herd module. However, this module replaces explicit within-host tracking with a calibrated, state-based approximation to scale with improved computational efficiency.

Disease progression for each agent is represented via independent draws from Weibull distributions estimating the duration of latent, preclinical, and clinical phases. A smooth sigmoid function maps time since infection to probabilities of being in infectious states, enabling classification of agents as susceptible, exposed, preclinical, clinical, or recovered at each time step. Within-farm transmission follows stochastic contact events conditioned on contact rates and fixed per-contact transmission probabilities. For the current study, a per-hour contact rate of 0.15 and a per-contact transmission probability of 0.2 were used.

A spatial network of farms is generated using configurable topologies (e.g., Erdős–Rényi, small-world, or geometric), with edge weights modulated by inter-farm distance to reflect the likelihood of cattle movement. Cattle are stochastically moved from non-quarantined farms to neighboring nodes at fixed intervals. Movement events serve as the primary mechanism for inter-farm transmission.

To incorporate surveillance and control, the model triggers quarantine upon detecting the first clinical case within a farm. A configurable delay of twenty-four hours precedes quarantine enforcement, after which the farm ceases all outbound movement. Quarantine status persists for the duration of the simulation. This framework enables testing of detection-based interventions and their effects on outbreak dynamics.

*Model calibration and simulation design* Model calibration was conducted by minimizing the sum of squared differences between simulated viral load trajectories and empirical data from the empirical animal transmission study. Calibrated parameters were used to conduct 10,000 independent trials of the room-to-room simulation (18 cattle per trial; 180,000 total agents), and 1000 simulations for each of two within-herd scenarios (100,000 total agents per scenario). These scenarios differed only in whether preclinical transmission was permitted. All other parameters remained constant.

The between-farm module was parameterized using outputs from the within-herd simulations. State probabilities for infectiousness were derived from phase durations under each within-herd scenario. A total of 4000 between-farm simulations were conducted, 2000 for each scenario, using identical network structures, movement patterns, and quarantine configurations to isolate the effects of preclinical infectiousness. Network sizes ranged from 20 to 1000 farms, each with 20 to 500 cattle.

## Data Availability

Code and data for the empirical experiment and simulations presented in this manuscript have been archived on the Open Science Framework (https://doi.org/10.17605/OSF.IO/P6XKE, [https://osf.io/qf2wr/] and are available on GitHub ([https://github.com/geoepi/fmdv-preclinical]).
